# Soluble TREM-1 plasma levels are associated with acute kidney injury, acute atrial fibrillation and prolonged ICU stay after cardiac surgery- a proof-concept study

**DOI:** 10.3389/fcvm.2023.1098914

**Published:** 2023-07-13

**Authors:** Marie Vandestienne, Rayan Braik, Jean-Rémi Lavillegrand, Geoffroy Hariri, Zoe Demailly, Nadine Ben Hamouda, Fabienne Tamion, Thomas Clavier, Hafid Ait-Oufella

**Affiliations:** ^1^ Centre de Recherche Cardiovasculaire de Paris (PARCC), Université de Paris, Inserm U970, Paris, France; ^2^ Service de Réanimation Chirurgicale, Assistance Publique – Hôpitaux de Paris, Groupe Hospitalier Pitié-Salpétrière, Institut du Coeur, Paris, France; ^3^ Université de Normandie, UNIROUEN, Inserm U1096, FHU REMOD-VHF, Rouen, France; ^4^ Service D’anesthésie-Réanimation Chirurgicale, CHU De Rouen, Rouen, France; ^5^ Service D’Immunologie, Hôpital Européen Georges Pompidou, Assistance Publique – Hôpitaux de Paris, Paris, France; ^6^ Service de Médecine Intensive-Réanimation, CHU De Rouen, Rouen, France; ^7^ Service de Médecine Intensive-Réanimation, Assistance Publique – Hôpitaux de Paris, Sorbonne Université, Paris, France; ^8^ Hôpital Saint-Antoine, Sorbonne Université, Paris, France

**Keywords:** cardiopulmonary bypass, cytokine, TREM-1, inflammation, outcome

## Abstract

**Background:**

Cardiopulmonary bypass (CPB) during cardiac surgery leads to deleterious systemic inflammation. We hypothesized that TREM-1, a myeloid receptor shed after activation, drives systemic inflammation during CPB.

**Methods:**

Prospective observational bi-centric study. Blood analysis (flow cytometry and ELISA) before and at H2 and H24 after CPB. Inclusion of adult patients who underwent elective cardiac surgery with CPB.

**Results:**

TREM-1 expression on neutrophils decreased between H0 and H2 while soluble (s)TREM-1 plasma levels increased. sTREM-1 levels increased at H2 and at H24 (*p* < 0.001). IL-6, IL-8, G-CSF and TNF-α, but not IL-1β, significantly increased at H2 compared to H0 (*p* < 0.001), but dropped at H24. Principal component analysis showed a close relationship between sTREM-1 and IL-8. Three patterns of patients were identified: Profile 1 with high baseline sTREM-1 levels and high increase and profile 2/3 with low/moderate baseline sTREM-1 levels and no/moderate increase overtime. Profile 1 patients developed more severe organ failure after CPB, with higher norepinephrine dose, higher SOFA score and more frequently acute kidney injury at both H24 and H48. Acute atrial fibrillation was also more frequent in profile 1 patients at H24 (80% vs. 19.4%, *p* = 0.001). After adjustment on age and duration of CPB, H0, H2 and H24 sTREM-1 levels remained associated with prolonged ICU and hospital length of stay.

**Conclusions:**

Baseline sTREM-1 levels as well as early kinetics after cardiac surgery identified patients at high risk of post-operative complications and prolonged length of stay.

## Introduction

Every year, several thousands of patients undergo cardiac surgery in the world (1). Despite improvements in minimally invasive and endovascular methods, cardiac surgery with cardiopulmonary bypass (CPB) remains the most frequently used procedure, but this is associated with life-threatening complications (2–4). Postoperative complications after cardiac surgery with CPB remain quite high, around 15%, including elevated mortality (5).

Surgery procedure may account for complications, but CPB by itself may also be responsible for tissue damage and organ failure due to the release of pro-inflammatory cytokines by circulating leucocytes and oxidative stress in response to both ischemia and exposure to extra-corporeal artificial surface (6, 7). Plasma levels of cytokines after CPB correlate with postoperative complications (8, 9). Despite improvement in the understanding of this CPB-induced “cytokine storm”, the engaged signaling pathways have not yet been fully elucidated, and no anti-inflammatory targeted therapy is available. We hypothesized that TREM-1, a receptor expressed by circulating neutrophils and monocytes may be implicated in CPB-induced inflammatory responses.

TREM-1, for *Triggering receptor expressed on myeloid cells* 1, is a member of the immunoglobulin “superfamily” containing a single variable-type immunoglobulin domain, which is broadly expressed on myeloid cells. Engagement of TREM-1, after association with the adapter protein DAP12 (which contains an immunoreceptor tyrosine-based activation motif), triggers a signaling pathway involving ZAP70, SyK, PI3 kinase, PLC-***γ***, and MAP kinases (10). Activation of these pathways leads to intracellular calcium mobilization, actin cytoskeleton rearrangement, and activation of transcription factors, including NF-*κ*B. In mice, engagement of TREM-1 with monoclonal agonist antibodies has been shown to promote the production of multiple pro-inflammatory cytokines and chemokines, including IL-8, CCL2, CCL3, GM-CSF (11, 12), as well as stimulating rapid neutrophil degranulation and oxidative burst (13). Several studies have shown that TREM-1 participates in inflammation-induced organ damage in sepsis through cooperation with TRL-4 (13, 14). More recently, its role in acute and chronic sterile inflammation has been reported, including in acute myocardial infarction (MI) and atherosclerosis, through the regulation of cytokine production and myeloid cell trafficking (15, 16). One of the features of TREM-1 is the release of a soluble form of the receptor after stimulation (17). Several human studies have shown that plasma levels of sTREM-1 could be used as diagnosis and prognosis markers for severe infections. Our group has previously reported that sTREM-1 is an powerful predictive factor of 2-year mortality or MI recurrence after acute MI (15).

The aim of our study was to assess: 1/the kinetics of TREM-1 expression in circulating myeloid cells, 2/ the relationship between sTREM-1 and pro-inflammatory cytokines plasma levels, and 3/ the association between sTREM-1 plasma levels and postoperative outcome.

## Methods

### Study scheme

A first prospective pilot study was conducted in the intensive Care Unit (ICU) of cardiac Surgery department in a tertiary teaching hospital (Pitié-Salpétrière Hospital, Paris). Patients older than 18 years of age, eligible for cardiac surgery with CPB of more than 1 h were included. A second prospective observational study was conducted between June 2018 and April 2019 in the ICU of cardiac Surgery in another tertiary teaching hospital (Charles Nicolle Hospital, Rouen) (18)*.* Patients older than 18 years of age, eligible for cardiac surgery with CPB of more than 1 h were included. Patients under guardianship, pregnant women, patients undergoing an emergency surgical procedure, and patients with a planned CBP duration of less than 1 h were excluded. Several exclusion criteria have been defined: chronic auto-immune or inflammatory diseases, active cancer, immunocompromised patients, chronic corticosteroid treatment.

Anaesthesic induction was achieved by the combination of hypnotic (propofol or etomidate) and morphinic (Sufentanil or Remifentanil) drugs. Maintenance of anaesthesia was done with propofol. A bolus of heparin was administered intravenously before the start of CPB and antagonized by protamine sulfate at the end of the procedure. Cardioplegia was performed with either hyperkalemic solution enriched with beta-blocker or by Custodiol. Mean arterial blood pressure was maintained between 50 and 70 mmHg. Other therapies were left to the choice of the clinician in charge of the patient.

### Data collection

For each patient, sex, age and body mass index (BMI), co-morbidities, duration of CPB, type and duration of surgery were collected. Requirement of vasopressor and invasive mechanical ventilation were evaluated 2 and 24 h (H2 and H24) after the end of CPB. Acute kidney injury (AKI) was assessed by the kidney disease improving global outcomes score (KDIGO) classification (19) at H24.

### Flow cytometry

Leukocyte suspension was obtained from blood and extracellular antigens were stained with fluorescent-labeled anti-human antibodies (See below) for 30 min at 4°C. Flow cytometric acquisitions were done on a BD LSRFortessa (BD Biosciences), and data were analyzed using FlowJo Software (TreeStar, Inc.). Forward scatter (FSC) and side scatter (SSC) were used to gate live cells, red blood cells, debris, cell aggregates, and doublets being excluded. In the blood, neutrophils were identified as CD66b+ living cells, and monocytes as CD66b-CD14+ cells expressing CD16 or not.

The following primary conjugated antibodies were used for the staining in blood (BioLegend®).

Pacific blueTM anti-human CD66b Clone G10F5; PE/Cyanine7 anti-human CD14 Clone 63D3; PerCP/Cyanine5.5 anti-human CD16 Clone 3G8; APC anti-human CD354 (TREM-1) Clone TREM-26.

### Cytokine and sTREM-1 dosage

Biological blood samples were all collected from the arterial cannula. Blood samples were taken just after anesthetic induction (H0) and then 2 and 24 h (H2 and H24) after the end of CPB. Blood samples were stored in EDTA tubes (4 ml) and then immediately centrifuged at 3000 G for 15 min. The plasma was then collected in microtubes and frozen at −80°C until analysis. Plasma concentrations of soluble TREM-1 (pg/ml) were determined in duplicate by enzyme linked immunosorbent assay (RnD Systems®) and the mean value was recorded. The levels of 5 cytokines/growth factor (IL-1β, IL-6, IL-8, TNF-α, G-CSF) were measured by Luminex technology according to the manufacturer's instructions (Bio-Plex, Bio-Rad, 5-Plex Assays panel, Marnes-la-Coquette, France).

### Statistical analysis

Data are expressed as mean (standard deviation), medians (IQRs) or proportion (%). The kinetics of cytokines and sTREM-1 were assessed at different times points by repeated measures ANOVA or a Friedman test. The comparison of mean or median values was performed by applying a pairwise comparison test of Mann-Whitney, Welch, Wilcoxon or Kruskal-Wallis Rank Sum Test, depending on the distribution of variables and equality of variances. For categorical variables, Fisher's exact test or the Chi-square test were used. To study the similarity of the patients according to the level of sTREM-1, a hierarchical classification was carried out using the package “pheatmap” (20). The relationship between cytokines was evaluated by applying a Pearson correlation matrix using the “rstatix” package (21). Principal component analysis (PCA) and multidimensional scaling (MDS) were applied on the basis of the results of the matrix correlation using the “FactorMineR” package (22, 23). The relationship of sTREM-1 with other cytokines was performed by applying a focused principal component analysis, according to the method of Falissard et al. (24) using the “Psy” package (25). The predictive capacity of s-TREM1 was evaluated by a ROC curve using “pROC” and “verification” packages (26, 27). Best cut-off for sensitivity and specificity was calculated by Youden index. Kaplan-Meier curves were used to assess the relationship between sTREM-1 and time to ICU/hospital discharge using the package “survival” (28). The comparison of curves was done by log-rank test and adjustment was done by Cox proportional-hazards model. Prolonged ICU stay and prolonged hospital length of stay were defined as duration higher than the third quartile (5 and 18 days, respectively). A sTREM-1 level higher than the third quantile was defined as “high”. Acute renal failure was defined according to the KDIGO classification as a score higher than 1 (19).

The significance level of 5% of the *p*-value was retained. Statistical adjustments of the *p*-value were done by the Holm method (29). All statistical tests were performed using the free software R version 4.0.3 (30).

### Ethics and consent

The pilot study was approved by the ethics committee of the French Society of Intensive Care medicine (CE SRLF 21-91, October 10th 2021). The second study (N°2017/179/HP) was approved by the South Mediterranean II Ethics Committee (n° CPP 2017-A03375-48, obtained April 18th 2018), both being in accordance with French legislation and the ethical principles of the Declaration of Helsinki. All patients included in the present study expressed their written consent.

## Results

### Flow cytometry study

**Figure 1:**
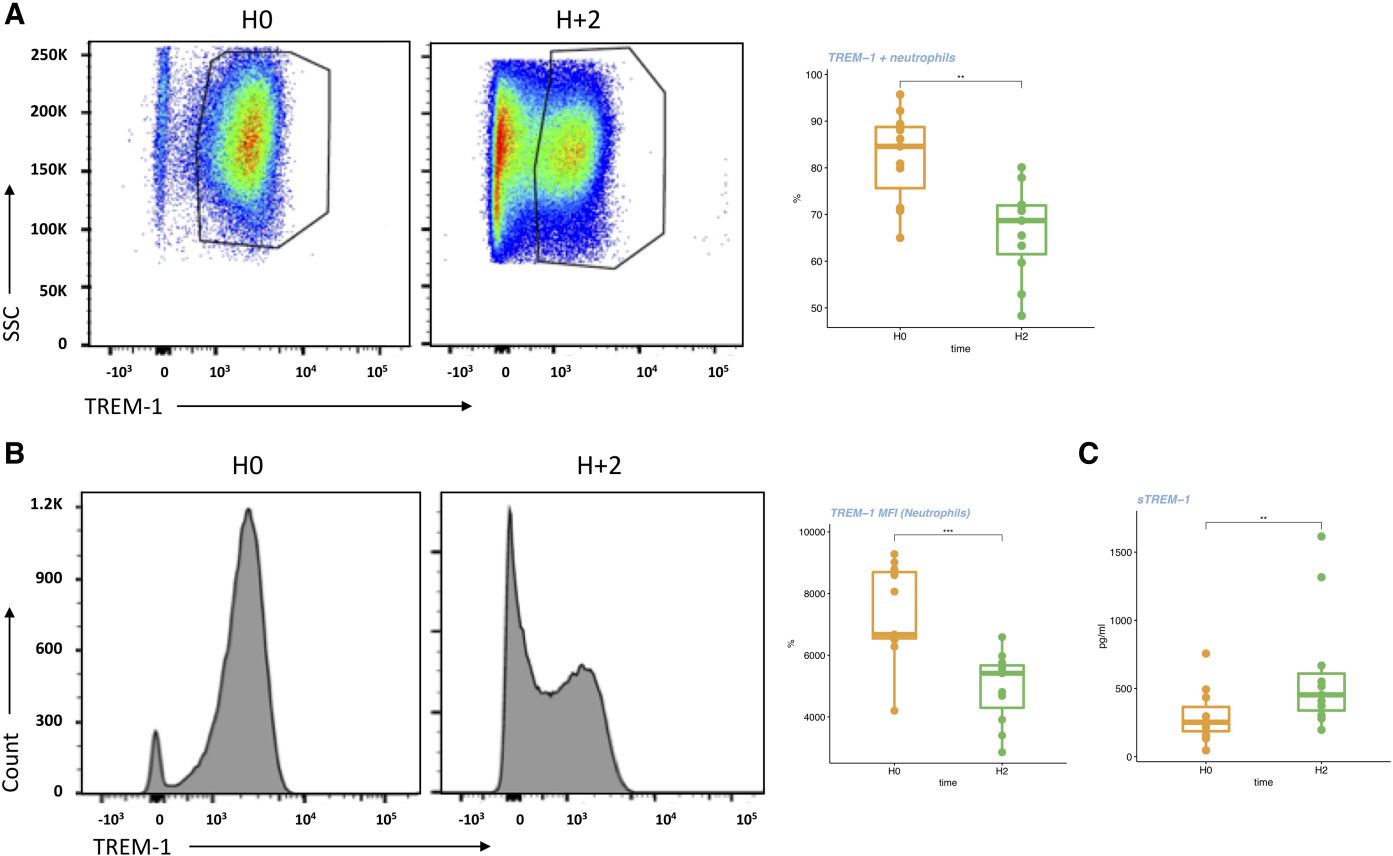
Membrane and soluble TREM-1 early changes during CPB (pilot study). (**A**) Flow cytometry analysis and representative dot plots of TREM-1 expression (%) on circulating neutrophils. (**B**) Flow cytometry analysis and representative pictures of TREM-1 expression (MFI) on circulating neutrophils. (**C**) s-TREM1 plasma levels at H0 and H2. For the box-and-whisker plots, the lower and upper borders of the box represent the lower and upper quartiles (25th percentile and 75th percentile). The middle horizontal line represents the median. The lower and upper whiskers represent the minimum and maximum values. Paired non-parametric test, **, *p* < 0.001,***, *p* < 0.001.

First, in a pathophysiological study including 11 patients (Supplementary Table S1), we aimed to analyze kinetic of membrane TREM-1 on different circulating myeloid subsets as well as the kinetic of plasma soluble form. Using flow cytometry, we found that TREM-1 expression on neutrophils significantly decreased between H0 and H2 after CPB (% of TREM-1 + neutrophils: 89 (85–96) vs. 69 (60–72), respectively, *p* < 0.004; and Mean Fluorescent Intensity: 6674 (6502–8794) vs. 5416 (3910–5762) %, respectively, *P* = 0.005) (Figures 1A,B; Supplementary Figure S1A) while TREM-1 expression on monocytes did not change (Supplementary Figures S1B,C). Conversely, between H0 and H2, plasma levels of sTREM-1 significantly increased (253 [157–434] vs. 454 [305–668] pg/ml, respectively, *p* < 0.005) (Figure 1C).

### Second prospective cohort- patients’ characteristics

Next, in a second larger cohort, we evaluated 1/the relationship between changes in sTREM-1 and cytokine levels, and 2/the relationship between sTREM-1 levels and patients’ outcome. Forty-six patients were included. Their main characteristics are shown in Table 1. The median age was 68 (11) years, mainly male (65%) and SAPS II was 34 (10). Heart surgery was done for valve disease (56.5%), coronary artery diseases (19.6%) or both (23.9%). Mean duration of CPB was 121 (40) minutes. At H24, 13 patients (28.3%) required support organ therapy including vasopressor infusion (*n* = 9, 19.6%) and mechanical ventilation (*n* = 4, 8.7%), and 15 patients developed acute atrial fibrillation (32.6%). At H48, 7 patients developed acute kidney failure (KDIGO > 1, 15.2%). No death was recorded. The mean length of stay was 5.4 (6.1) days in ICU and 17.6 (15.0) days in hospital.

**Table 1:** Characteristics of patients (2nd cohort).

Demographic characteristics	Value
Number of patients (*n*)	46
Age [years, mean (SD)]	68 (11)
Gender (F/M)	16/36
BMI [kg/m^2^, mean (SD)]	28.17 (4.7)
Hypertension (*n*, %)	29 (63.0)
Diabetes (*n*, %)	10 (21.7)
Chronic respiratory disease (*n*, %)	5 (10.8)
History of atrial fibrillation (*n*, %)	7 (15.2)
Biological characteristics	
Leukocytes (Giga/L) [mean (SD)]	6.97 (1.79)
Neutrophils (Giga/L) [mean (SD)]	4.44 (1.69)
Creatinine (micromol/L) [mean (SD)]	80 (22)
C-Reactive protein (mg/L) [mean (SD)]	5 (6)
Per-operative characteristics	
Type of surgery [*n* (%)]:	
-CABG	9 (19.6)
-Valve surgery	26 (56.5)
-CABG + valve surgery	11 (23.9)
Duration of surgery [min, mean (SD)]	223.2 (53.7)
Duration of CPB [min, mean (SD)]	121.4 (40.0)
Post-operative characteristics	
SAPS II [score, mean (SD)]	34.2 (10.1)
Vasopressor H24 [*n* (%)]	9 (19.6)
Mechanical ventilation H24 [*n* (%)]	4 (8.7)
Acute kidney injury [KDIGO > 1, [*n* (%)]	4 (8.7)
Length of ICU stay [days, mean (SD)]	5.3 (6.1)
Length of hospital stay [days, mean(SD)]	17.63 (15.0)
Death [*n* (%)]	0 (0)

This table represents the pre, per and post-operative characteristics of the patients. The values are expressed as mean ± standard deviation (SD), number (*n*) and percentage (%).

F, female; M, male; CABG, coronary artery bypass graft; Min, minutes; CPB, cardiopulmonary by-pass; ICU, intensive care unit; KDIGO, kidney disease international outcomes score; SAPS II, simplified acute physiology score.

### Kinetic of plasma cytokines and sTREM-1

**Figure 2:**
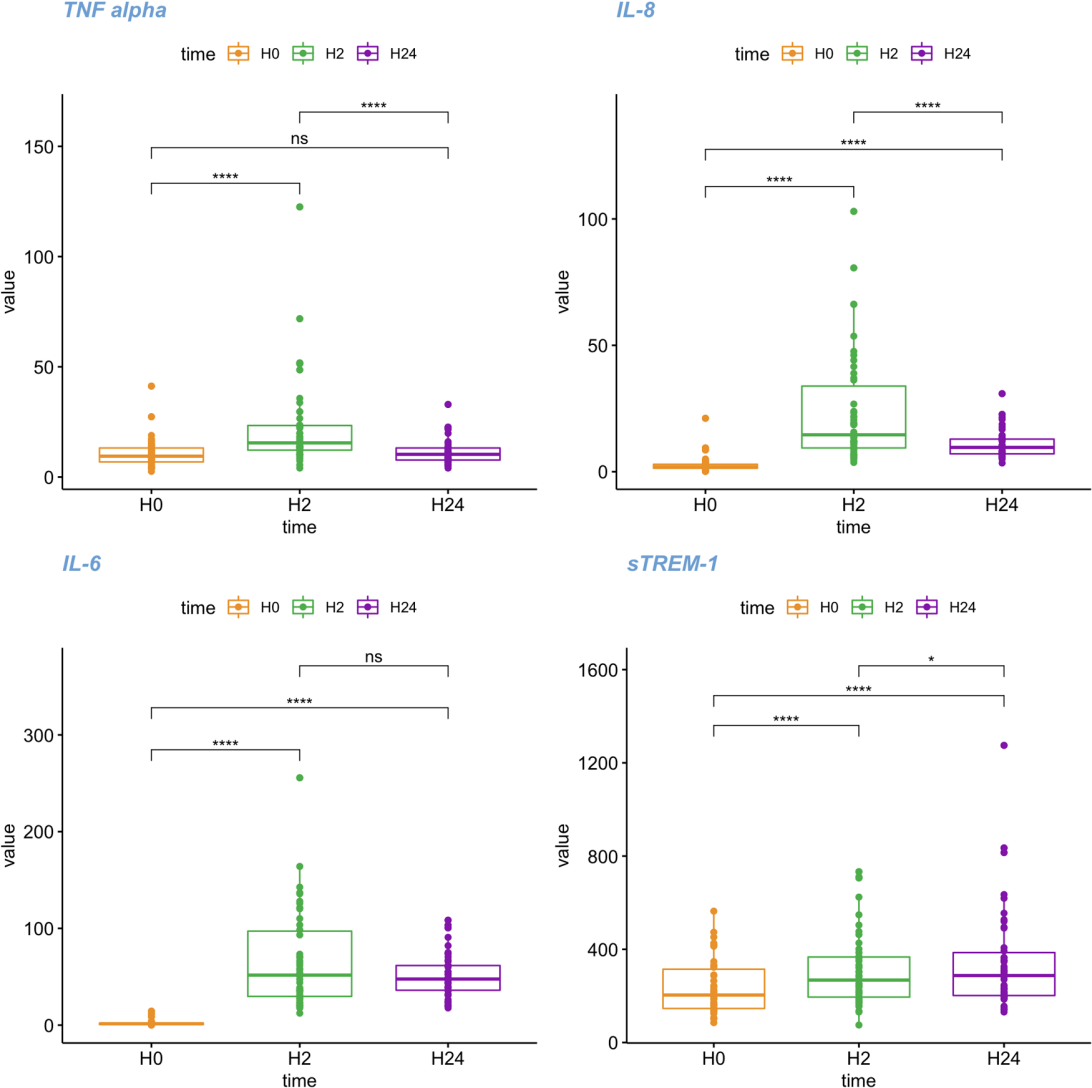
Kinetics of sTREM-1 and cytokine plasma levels over time. Boxplot representing the kinetics of s-TREM1, IL-6, IL-8 and TNF-α levels, at different time points (H0: first sampling, immediately after anaesthetic induction, Orange, H2: two hours after the end of CPB, Green, H24: 24 h after the end of CBP, Purple). For the box-and-whisker plots, the lower and upper borders of the box represent the lower and upper quartiles (25th percentile and 75th percentile). The middle horizontal line represents the median. The lower and upper whiskers represent the minimum and maximum values. *: adjusted *p* value (Holm) lower < 0.05 pairwise comparison with a Wilcoxon test. ** <0.01; ***: adjusted *p* value (Holm) lower < 0.001; ****: adjusted *p* value (Holm) lower < 0.0001.

During CPB, we observed significant changes in IL-6, IL-8, G-CSF, TNF-α and sTREM-1 plasma levels (Friedman test, *p*.adjust < 0.001 for all biomarkers), but no changes in IL-1β (*p* = 0.671) (Figure 2; Supplementary Figure S2). Between H0 and H2, plasma levels of sTREM-1 and all other measured cytokines, except IL-1β, significantly increased (paired test, *p*.adjust < 0.001). Between H2 and H24, sTREM-1 levels further increased (p.adjust = 0.019), whereas levels of IL-8, G-CSF, TNF-α decreased and IL-6 remained stable (Figure 2; Supplementary Figure S2). sTREM-1 levels at H2 and H24 correlate with CPB duration (*r* = 0.37, *p* = 0.011 and *r* = 0.35, *p* = 0.019 respectively) (Supplementary Figure S3).

**Figure 3:**
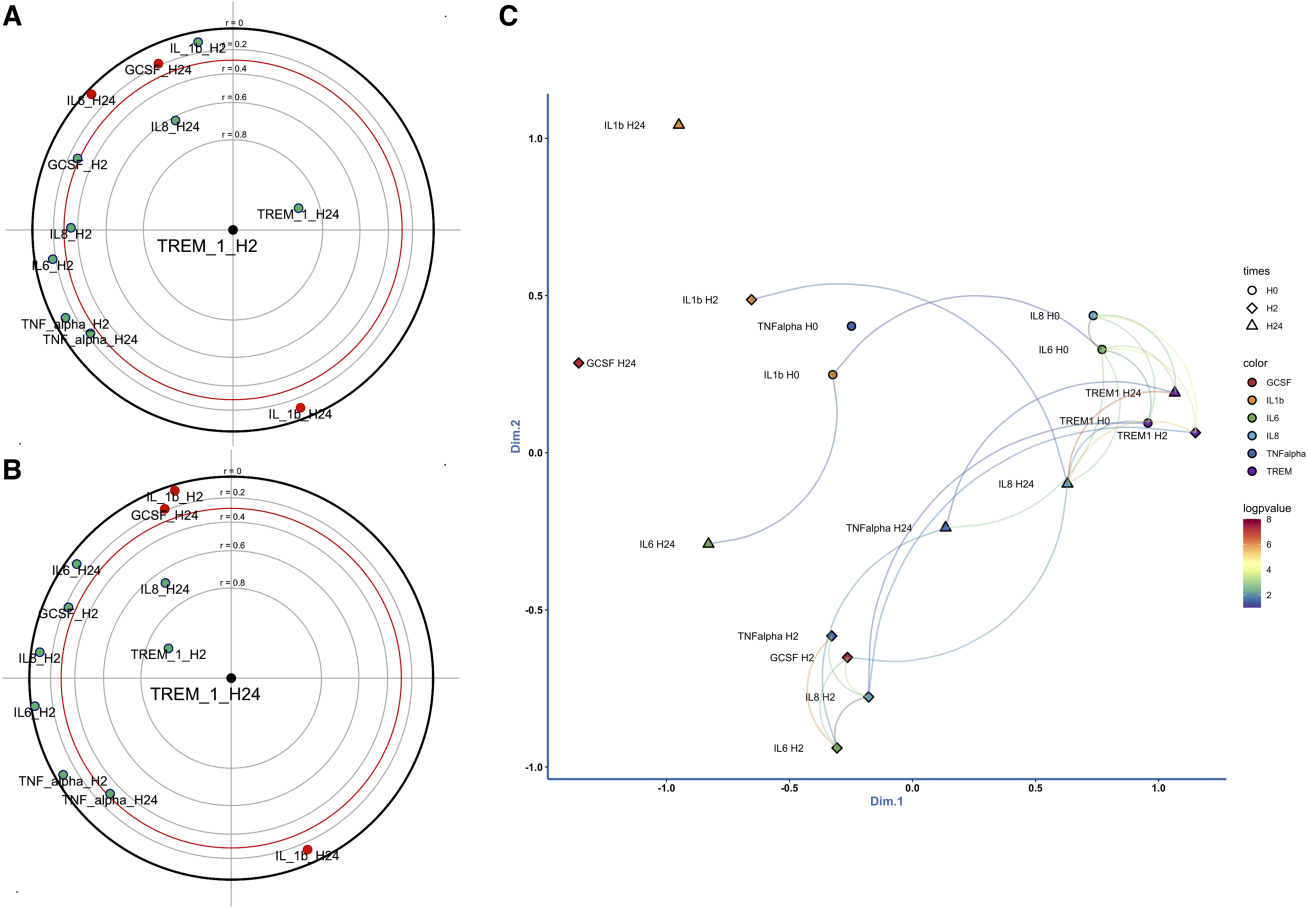
Correlation between sTREM-1 levels and cytokines at H2 (**A**) and H24 (**B**). Dimension reduction of the correlation matrix by performing a principal component analysis focused on sTREM-1 levels at H2 and H24. Correlated variables are close or diametrically opposite (for negative correlations), independent variables make a right angle with the origin. r: correlation coefficient. (**C**), two-dimensional representation of the correlation matrix. Two-dimensional representation of the correlation matrix by applying a multidimensional scaling metric. The closer the elements of the graph are, the higher the value of their correlation. The significant interactions are drawn by a curve (*p* < 0.01). A color palette is used to represent the certainty of association by the logarithm of 10 of the *p* values.

H2 sTREM-1 levels correlated with all measured cytokines, except IL-1β, the correlation being highly significant with IL-8 [H24, *r* = 0.62 (95% CI = 0.18–0.85), p.adjust = <0.0001]. H24 sTREM-1 levels were also significantly correlated with H24 IL-8 [*r* = 0.67 (95% CI = 0.26–0.87), p.adjust = <0.0001] (Figures 3A,B**)**. Multivariate analysis by FPCA confirmed the strong relationship between sTREM-1 and IL-8 levels (Figure 3C). MDS applied to the matrix correlation showed two aggregated groups of biomarkers. One group included TNF-α, IL-8, IL-6 and G-CSF at H2, and another group IL-8 and sTREM-1 (Supplementary Figure S4).

### Patient profiling using sTREM-1 kinetics

**Figure 4:**
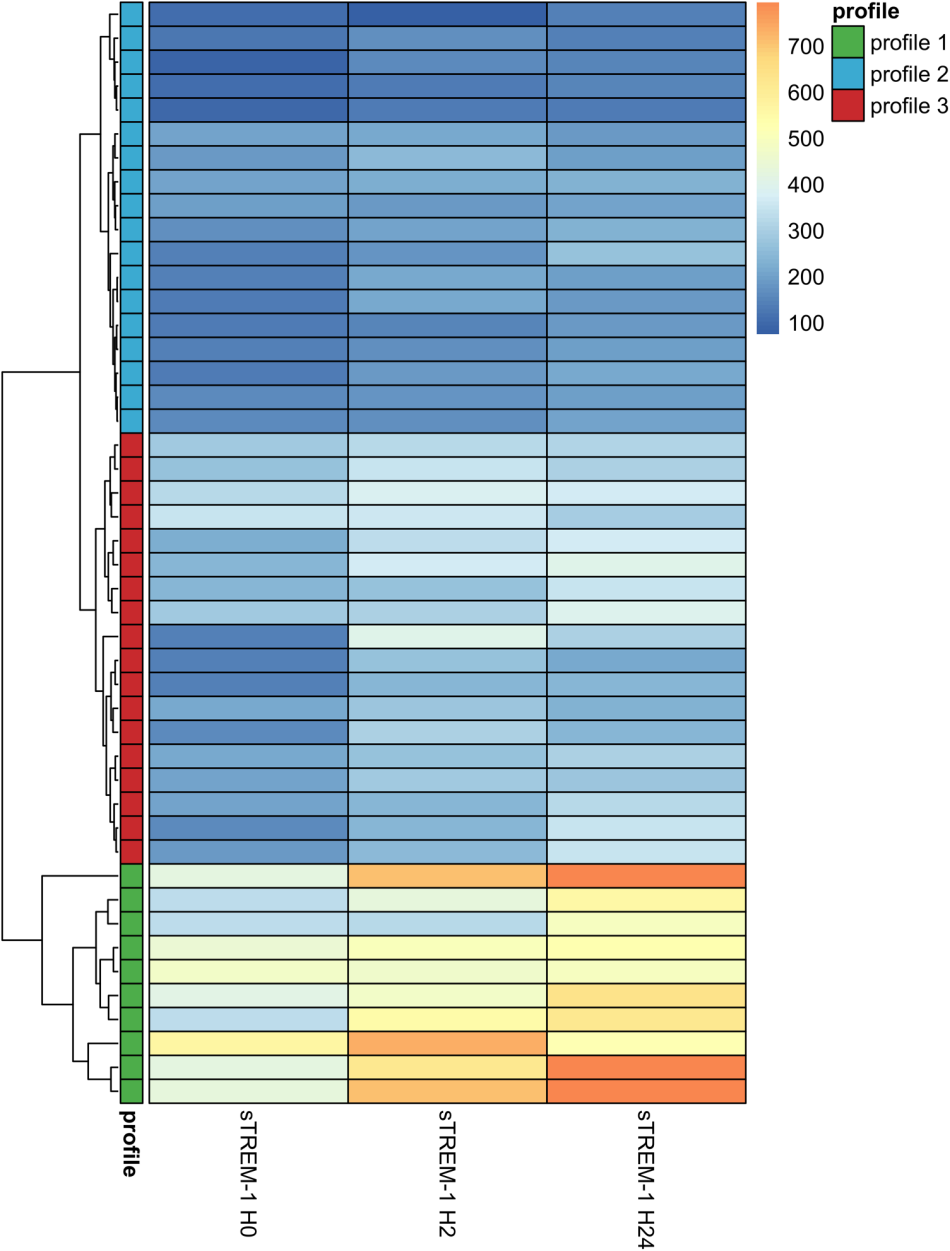
Heatmap representing for each patient the sTREM-1 value at 3 times points. Heatmap showing the raw values of sTREM-1 in pg/ml for each patient at different time points. The ascending hierarchical classification highlights three profiles. Profile 1 corresponds to patients with high baseline sTREM-1 levels associated with a strong increase between H0 to H24, profile 2 corresponds to patients with low baseline sTREM-1 levels which remain relatively stable between H0 and H24 and profile 3 with moderate baseline sTREM-1 levels which remain stable or increased slightly between H0 and H24.

Baseline sTREM-1 levels varied significantly between patients, as well as the kinetics after CPB. The hierarchical clustering allowed to first identify 2 different patient profiles “profile 1 and non-profile 1” and then among “non-profile 1” 2 subgroups named “profile 2 and profile 3” (Figure 4; Supplementary Table S2**)**. Profile 1 patients were characterized by high baseline levels of sTREM-1 and marked changes between H0, H2 and H24, Profile 2 patients had low baseline sTREM-1 levels that remained stable over time whereas profile 3 patients with moderate baseline sTREM-1 levels that remained stable or slightly increased over time.

**Figure 5:**
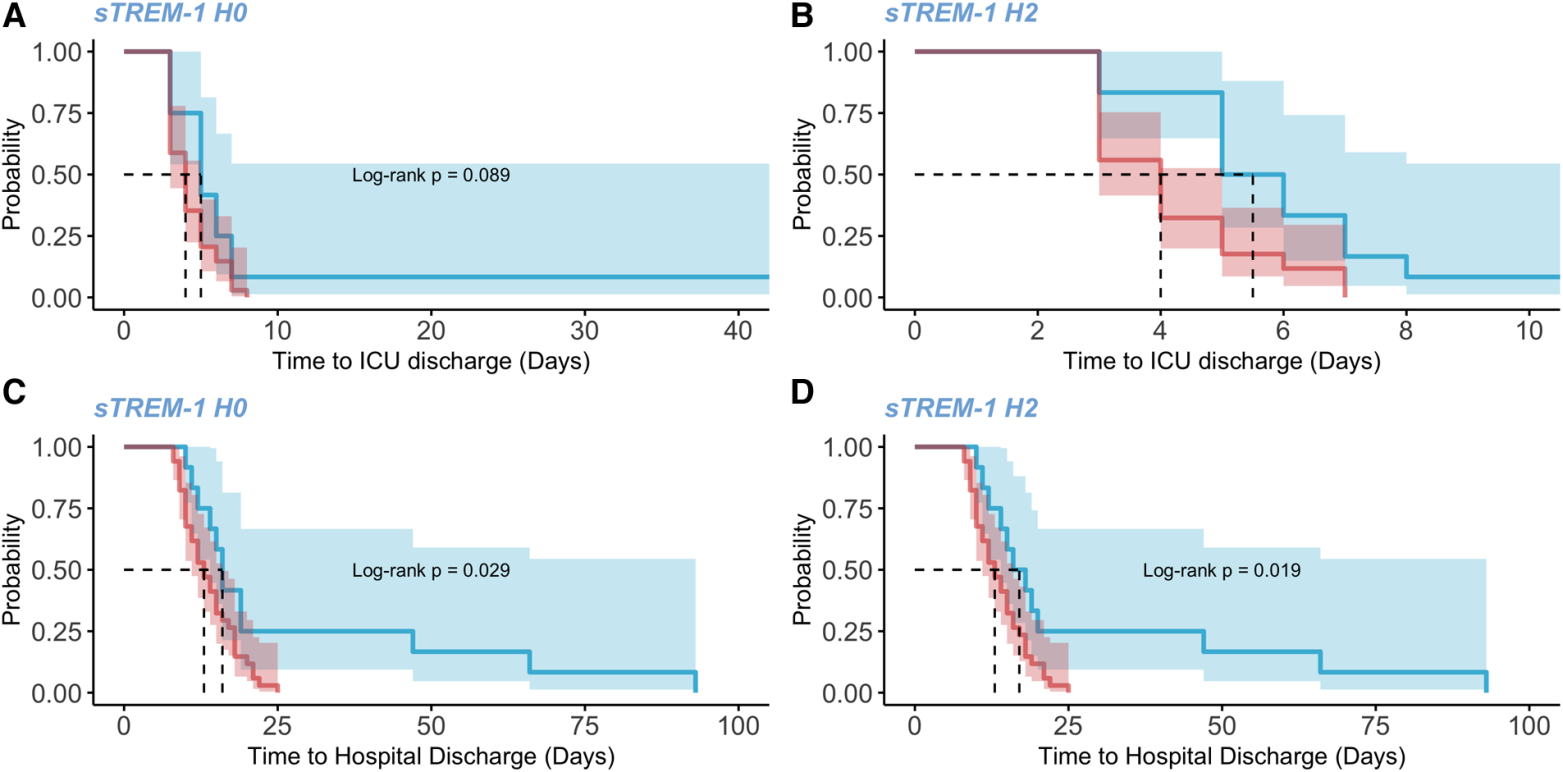
Length of hospital and ICU stays between patients with low and high sTREM-1 levels. Kaplan-Meir curves, representing the time to ICU and hospital discharge (days) between patients with low or high sTREM-1 levels (95% confidence interval). The curves are compared by a log-rank test. A high level of sTREM-1 is defined as a level higher than the third quantile in either cohort. (**A**) time to ICU discharge between patients with high or low levels of sTREM-1 at H0. (**B**) time to ICU discharge between patients with high or low levels of sTREM-1 at H2. (**C**) time to ICU discharge between patients with high or low levels of sTREM-1 at H24. (**D**) time to hospital discharge between patients with high or low levels of sTREM-1 at H0.

Clinical parameters according to sTREM-1 profiles are depicted in Table 2; Supplementary Table S2. Duration of both CPB and surgery was not different between groups but profile 1 patients were older. However, when compared to profile 2 or 3 (named 2/3), profile 1 patients developed more severe organ failure after CPB, requiring higher norepinephrine dose at H24 (0.60 ± 0.16 vs. 0.11 ± 0.03 µg/kg/min, *p* = 0.044), and having higher SOFA score (3.1 ± 3.8 vs. 1.0 ± 1.5, *p* = 0.011) and more frequently acute kidney injury at both H24 (30% vs. 3%, *p* = 0.039) and H48 (60% vs. 3%, *p* < 0.001). Finally, acute atrial fibrillation at H24 was more frequent in profile 1, compared to profile 2/3 (80% vs. 19%, *p* = 0.001). No significant difference was observed between profile 2 and profile 3 groups. Compared to profile 2/3, profile 1 patients had longer length of stay in both hospital and ICU (log rank *p* value = 0.024 and 0.025, respectively). After adjustment on age and CPB duration, the relationship between sTREM-1 levels and ICU length of stay remained statistically significant at H2 and H24. After adjustment on age and CPB duration, the relationship between sTREM-1 levels and hospital length of stay remained statistically significant at H0, H2 and H24 (Figure 5; Supplementary Figure S5**)**.

**Table 2:** Comparison of clinical parameters between profile 1 patients and profile 2/3 patients.

Parameters	Profile 1	Profile 2 or 3	*p* value
Numbers of patients, *n*	10	36	
Age [years, mean (SD)]	76.0 (5.1)	66.1 (11.2)	**0** **.** **006**
Gender [male, *n* (%)]	6 (60.0)	30 (83.3)	0.250
BMI [kg·m^2^, mean (SD)]	30.13 (3.8)	27.63 (4.9)	0.099
Blood cardioplegia [*n* (%)]	3 (33.3)	16 (44.4)	0.647
Duration of CPB [min, mean (SD)]	133.90 (52.2)	118.00 (36.0)	0.448
Duration of surgery [min, mean (SD)]	224.90 (66.3)	222.83 (50.8)	0.915
Norepinephrine H24 [µg/kg/min, mean (SD)]	0.60 (0.16)	0.11 (0.03)	0.072
Dobutamine H24 [µg/kg/min, mean (SD)]	0.47 (1.75)	0.75 (1.62)	0.259
Assisted ventilation H24 [*n* (%)]	2 (20.0)	2 (5.6)	0.424
AKI H24 [*n*(%)]	3 (30.0)	1 (2.8)	**0**.**039**
AKI H48 [*n*(%)]	6 (60)	1 (3)	**<0**.**001**
Atrial fibrillation H24 [*n*(%)]	8 (80)	7 (19.4)	**0**.**001**
SOFA H48 [mean (SD)]	3.1 (3.8)	1.0 (1.5)	**0**.**011**
Duration of ICU stay [days, mean (SD)]	9.40 (12.5)	4.25 (1.5)	**0**.**008**
Duration of hospital stay [days, mean (SD)]	30.70 (28.4)	14.00 (4.5)	**0**.**060**
sTREM-1, H0 [median (1st-3rd IQRs)]	418 [352–446]	168 [143–217]	**<0**.**001**
sTREM-1, H2 [median (1st-3rd IQRs)]	526 [466–685]	239 [181–292]	**<0**.**001**
sTREM-1, H24 [median (1st-3rd IQRs)]	587 [521–770]	232 [198–310]	**<0**.**001**

Bold values denote statistical significance at the *p* < 0.05.

Comparison of clinical parameters between profile 1 (increase in sTREM-1 levels) and profiles 2/3 patients (relative stabilization or decrease in sTREM-1 levels). Values expressed as mean ± standard deviation (SD) and as number (*n*) and percentage (%).

F, female; M, male; CABG, coronary artery bypass graft; Min, minutes; CPB, extracorporeal circulation; IGS2, prognostic score [reference]; KDIGO, hidney Ddsease international outcomes score^19^. Comparison using non-parametric test.

### Predictive value of sTREM-1

We compared the ability of sTREM-1 levels at H0, H1 and H24 and TREM-1 variations to predict prolonged stay (ICU and hospital), acute atrial fibrillation and acute kidney injury. Overall, we found that TREM-1 levels were better predictors than TREM-1 variations (Supplementary Table S3). TREM-1 levels measured at H0 and H2 had almost the same and the most important predictive value for post-operative complications. For example, the area under the curve of sTREM-1 levels at H0 to predict acute kidney injury was 0.83 (CI95%: 0.66–1, Se = 0.87, Sp = 0.86, cut-off = 325.8 pg/ml, *p* = 0.003). The ability of sTREM-1 values at H0 to predict prolonged length of stay in ICU (>5 days) was 0.73 (CI95%: 0.57–0.89, Se = 0.83, Sp = 0.56, cut-off = 191.0 pg/ml, *p* = 0.009) and to predict prolonged length of stay in hospital (>18 days) was 0.70 (CI95%: 0.50–0.90, Se = 0.92, Sp = 0.50, cut-off = 339.6 pg/ml, *p* = 0.016).

## Discussion

In two prospective cohorts of patients with elective cardiac surgery, we observed an early decrease of TREM-1 expression on circulating neutrophils, associated with an early sustained increase in soluble TREM-1 plasma levels after CPB. Levels of inflammatory cytokines increased at H2 but decreased later on. By performing a hierarchical clustering based on sTREM-1 kinetics, we were able to identify a group of patients who developed more frequent acute kidney injury and atrial fibrillation after CPB and had prolonged length of stay in both ICU and hospital.

Our findings on cytokine kinetics are consistent with previous studies showing an increase of TNF-α, IL-6, and IL-8 within minutes after the start of surgery (6, 31), changes being correlated with CPB and ischemia duration. Soluble TREM-1 levels correlated with pro-inflammatory cytokine levels, in agreement with previous gain- and loss-of-function experimental studies showing that TREM-1 engagement drives cytokine production through NF-*κ*B activation (32, 33). Here, mediators that stimulate TREM-1 remain unknown, but several candidates might be suggested. First, circulating endotoxin, detected during CPB, could stimulate TLR-4, which in turn may promote both TREM-1 expression and activation (13). Angiotensin II released during CPB could also activate TREM-1 through AT1R receptor (34). We found that neutrophils were the main cellular source of sTREM-1 at H2, but we cannot rule out that sTREM-1 was released from endothelial cells (35) or circulating monocytes (36) at later stages. We did not observe any significant changes in IL-1β during CPB, which is also consistent with previous studies (37). This result is not clearly understood, but could be due to intraoperative hypothermia, which affect intracellular metabolism of IL-1 (37). We observed a strong correlation between sTREM-1 and IL-8, but the correlations with other pro-inflammatory cytokines were weaker. This makes sense since IL-8 is mainly produced by neutrophils that highly express TREM-1, whereas IL-6 and TNF-α are also released by cells that do not express TREM-1, including lymphocytes and endothelial cells (36). Our results are consistent with previous works showing a correlation between IL-8 plasma levels and post-CPB inflammatory responses (38).

One study has previously reported increased sTREM-1 levels after cardiovascular surgery in patients with systemic inflammatory response syndrome but the relationship between sTREM-1 variations and outcome remained unknown (39). Based on sTREM-1 kinetics, we identified a group of patients at high risk for acute kidney injury and acute atrial fibrillation after CPB. The association between sTREM-1 levels and acute kidney injury has been reported in septic shock, but the pathophysiological link between this receptor activation and kidney dysfunction remains unknown. We speculated that TREM-1 may promote kidney damage through the stimulation of proinflammatory cytokine production, as well as induction of oxidative stress (40, 41), or through chemokine production, which in turn orchestrates the recruitment of pathogenic immune cells in the kidney (42, 43). In addition, TREM-1 expressed by renal epithelial cells may promote kidney damage by inducing apoptosis and autophagy (44).

Profile 1 patients were characterized by higher vasopressor dose requirement at H24, which suggests that TREM-1 engagement potentiated post-CPB endothelial dysfunction and related impaired vasomotor tone. This is supported by a recent experimental study showing that TREM-1 regulates nitric oxide release by endothelial cells in sepsis, and mesenteric artery sensitivity to phenylephrine in ex vivo experiments (35). Unexpectedly, we observed that atrial fibrillation was more frequent in profile 1 patients, underlining the newly identified role of both local and systemic inflammation on atrial fibrillation occurrence (45–47).

In our study, we showed that high sTREM-1 levels were associated with prolonged ICU/hospital length of stay, which is a clinical relevant endpoint integrating several parameters such as post-operative complications. This association remained significant after adjustment for age and duration of CPB, but we cannot rule out that other confounders might impact on the relationship between sTREM-1 and post-operative complications. Overall, high sTREM-1 plasma levels help identifying patients with exaggerated systemic pro-inflammatory responses induced by CPB, responsible for cardiovascular system disorders, acute kidney injury and *in fine* prolonged length of stay.

Our results suggest that TREM-1, a master regulator of cytokine/chemokine production might be involved in the deleterious inflammatory response following CPB, even though a direct causative link cannot be established. TREM-1 inhibition could be an interesting strategy to be tested in this context to limit post-operative complications, such as kidney injury, and to shorten hospital length of stay. Our group and others have developed a pharmacological TREM-1 blocker, LR-12, which has been shown to provide benefits in experimental chronic diseases, including atherosclerosis (16) and aortic aneurysm (34), as well as in acute injury, including sepsis and acute MI (15). A phase IIa trial in septic shock patients has been recently conducted showing that TREM-1 blockade may be safe in critically ill patients (48). Based on our present findings, we believe that this targeted immunomodulatory strategy could be tested in the future in cardiac surgery patients. Given that H0 sTREM-1 levels are good predictor of post-operative outcome, we believe that pre-operative measurement of this soluble biomarker should be proposed to identify patients who would benefit of personalized preventive immune-modulatory treatment based on TREM-1 blockade.

Finally, we acknowledge some limitations. It is a bicentric study with a limited number of patients with no planned group size. Conclusions were drawn from two separate cohorts. Limited population size did not allow to evaluate the relationship between sTREM-1 and strong endpoints such as post-operative mortality. For all these reasons, further studies in larger population have to be performed to confirm our results, including more selected patients (ex: only coronary artery bypass graft) to limit confounders.

## Conclusion

In cardiac surgery, baseline sTREM-1 levels, as well as early variations, are associated with CPB-induced inflammation and post-operative complications, both being responsible for prolonged length of stay.

## Data Availability

The original contributions presented in the study are included in the article/**Supplementary Material**, further inquiries can be directed to the corresponding author.
