# Supplementary material for: Soluble TREM-1 plasma levels are associated with acute kidney injury, acute atrial fibrillation and prolonged ICU stay after cardiac surgery- a proof-concept study
Source: Front Cardiovasc Med. 2023 Jul 13;10:1098914. doi: 10.3389/fcvm.2023.1098914 (PMC10373879; doi:10.3389/fcvm.2023.1098914)
Supplement: Supplementary file 1 [file Datasheet1.pdf]

Supplemental digital content

Supplementary Table 1……………………………………Page 2

Supplementary Figure 1……………………..…………Page 3

Supplementary Figure 2……….……………………….Page 4

Supplementary Figure 3………..………………...……Page 5

Supplementary Figure 4………………………………Page 6

Supplementary Table 2……………….………………Page 7

Supplementary Figure 5………………………………Page 8

Supplementary Table 3……………..…………………Page 9

Supplementary Table 1

Characteristics	Value
Number of patients	10
Age [years, mean (SD)]	60 (15)
Gender (F/M)	3/7
BMI [kg/m^2, mean (SD)]	26 (3)
Hypertension (n)	4
Diabetes (n)	2
Euroscore 2 (median [1stIQR-3rdIQR])	1.83 [0.82-3.06]
Type of surgery [n (%)]:	
- CABG	3
- Valve surgery	3
- CABG + valve surgery	2
- Others	2
Duration of CBP minutes (median [1st-3rd IQRs])	133 [96-149]
TREM-1 H0, median [1st-3rd IQRs]	253 [157-434]
TREM-1 H0, median [1st-3rd IQRs]	454 [305-668]

Supplementary Table 1: pre-operative characteristics of the patients of the pilot study. The values are expressed as mean ± standard deviation (SD), number (n) and (median [1st-3rd IQRs]). F: female. M: male. CABG: coronary artery bypass graft. Min: minutes. CPB: cardiopulmonary by-pass.

Supplementary Figure 1

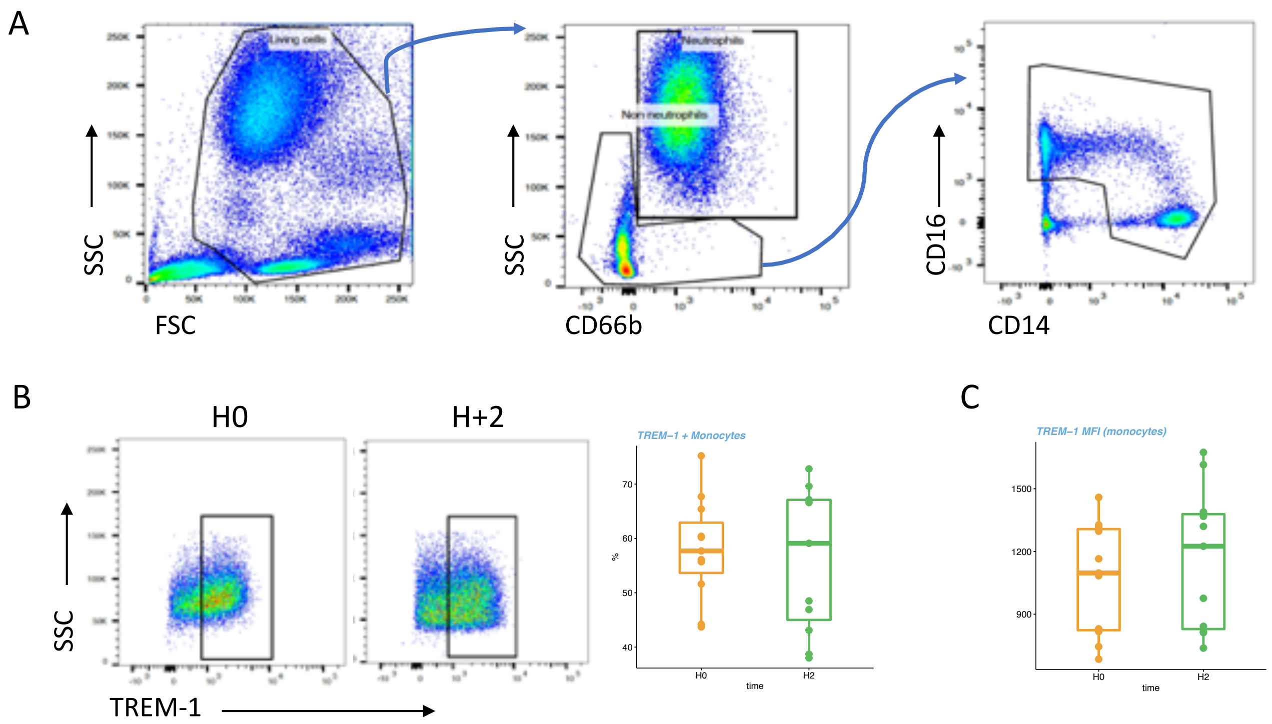

Supplementary Figure 1. Membrane and soluble TREM-1 early changes during CPB . A, Gating strategy by flow cytometry for myeloid cells in blood at baseline (H0, Orange) and 2 hours (H2, Green) after the end of CPB. Neutrophils were defined as CD66b+ cells among living cells and monocytes were defined as CD66b-CD16+CD14low and CD66b-CD16-CD14high cells. B, Flow cytometry analysis and representative dot plots of TREM-1 expression (%) on circulating monocytes. C, Flow cytometry analysis of TREM-1 expression (MFI) on circulating monocytes.

Supplementary Figure 2

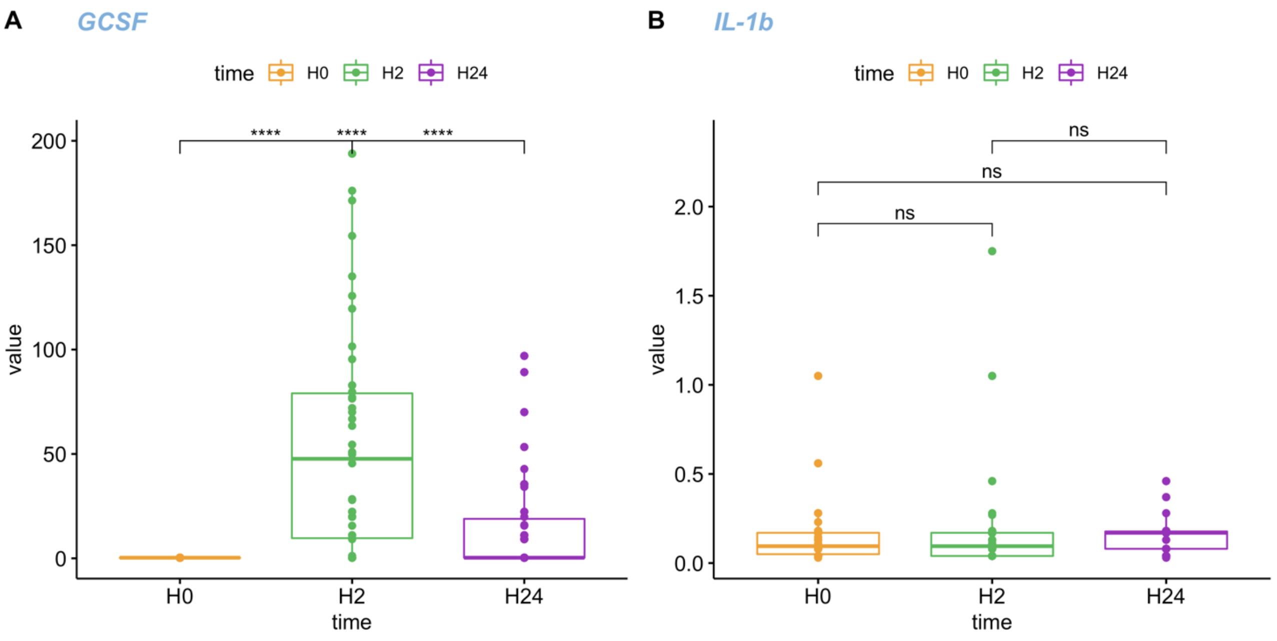

Supplementary Figure 2. Kinetics of sTREM-1 and cytokine plasma levels over time. Boxplot representing the kinetics of G-CSF and IL-1β levels, at different time points (H0: first sampling, immediately after anaesthetic induction, Orange, H2: two hours after the end of CPB, Green, H24: 24 hours after the end of CEC, Purple). For the box-and-whisker plots, the lower and upper borders of the box represent the lower and upper quartiles (25th percentile and 75th percentile). The middle horizontal line represents the median. The lower and upper whiskers represent the minimum and maximum values. ****: adjusted p value (Holm) lower < 0.001 pairwise comparison with a Wilcoxon test

Supplementary Figure 3.

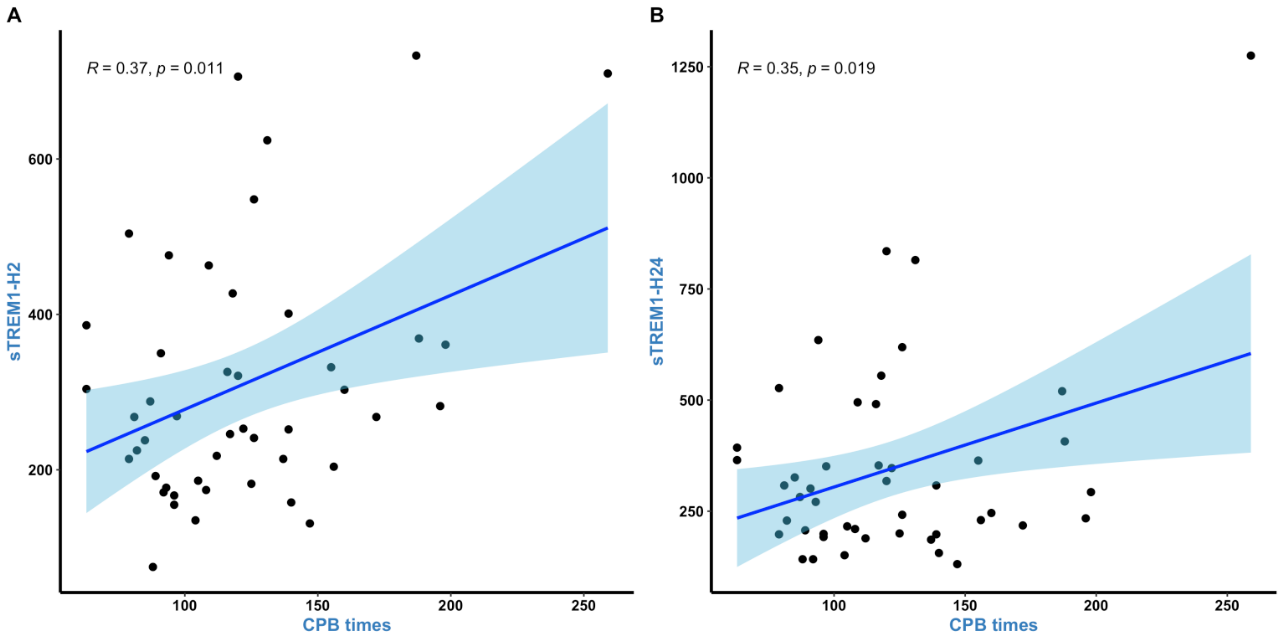

Supplementary Figure 3. Pearson correlation between sTREM-1 levels and CPB times (minutes).

Supplementary Figure 4

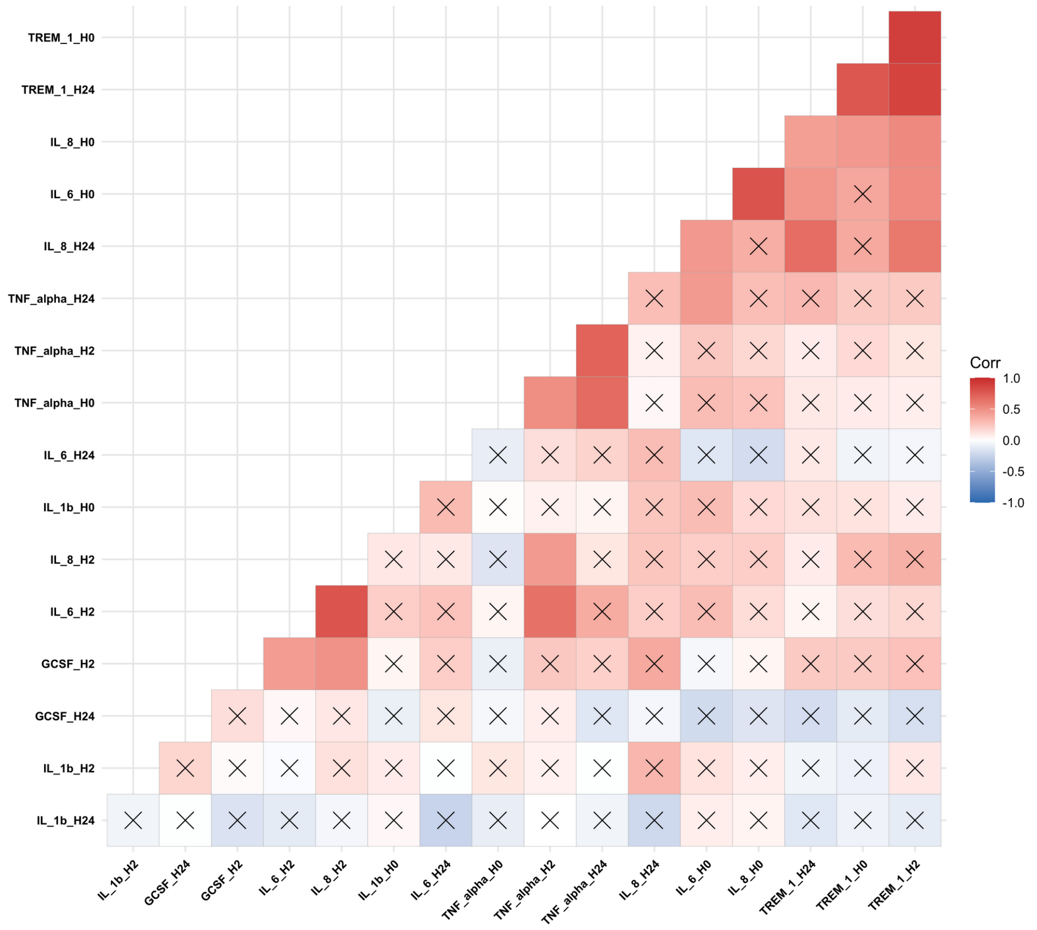

Supplementary Figure 4: Correlation matrix of all cytokines and sTREM-1 at different time points. Matrix correlation of all cytokines and sTREM-1. The crosses represent non-significant correlation (adjusted according to Holm's method). The value of the correlation coefficient (Pearson) is represented by a color gradient: blue (negative correlation), white (no correlation), red (positive correlation).

Parameters	Profile 1	Profile 2	Profile 3	p
n	10	18	18	
**AGE [years, mean (SD)]**	76.0 (5.1)	65.7 (11.8)	66.4 (10.9)	**0.022**
**Gender, [male, n (%)]**	6 (60.0)	13 (72.2)	17 (94.4)	0.077
**BMI [kg·m², mean (SD)]**	30.1 (3.8)	27.2 (5.3)	28.0 (4.5)	0.078
**Duration of CPB [minutes, mean (SD)]**	133.9 (52.2)	125.5 (44.4)	110.4 (24.1)	0.494
**Duration of surgery [minutes (mean (SD)]**	224.90(66.3)	217.4 (48.9)	228.3 (53.5)	0.890
**Norepinephrine H24 [µg/kg/min (mean (SD)]**	0.06 (0.16)	0.01 (0.04)	0.00 (0.01)	0.170
**Dobutamine H24 [µg/kg/min, mean (SD)]**	0.75 (1.62)	0.22 (0.73)	0.72 (2.37)	0.473
**Mechanical ventilation H24, n (%)**	2 (20.0)	2 (11.1)	0 (0.0)	0.178
**AKI H24, n (%)**	3 (30.0)	1 (5.6)	0 (0.0)	**0.022**
**AKI H48, n (%)**	6 (60.0)	0 (0.0)	1 (5.6)	**<0.001**
**Atrial fibrillation, n (%)**	8 (80.0)	5 (27.8)	2 (11.1)	**0.001**
**SOFA Day 2 [mean (SD)]**	3.1 (3.8)	1.3 (2.0)	0.8 (0.6)	**0.035**
**ICU lenght of stay [days, mean (SD)]**	9.4 (12.6)	4.5 (1.7)	4.00(1.2)	**0.024**
**Hospital lenght of stay [days, mean (SD)]**	30.7 (28.5)	14.8 (4.4)	13.2 (4.6)	0.089
**TREM-1 H0, median [1st-3rd IQRs]**	418 [352-446]	218 [169-260]	142 [126-170]	**<0.001**
**TREM-1 H2, median [1st-3rd IQRs]**	526 [466-685]	295 [268-345]	179 [160-211]	**<0.001**
**TREM-1 H24, median [1st-3rd IQRs]**	587 [522-770]	313 [285-352]	198 [163-209]	**<0.001**

Supplementary Table 2: Characterization of 3 groups of patients according to their sTREM-1 profile. Values expressed as mean ± standard deviation (SD), median [1st-3rd IQRs], number (n) and percentage (%). AKI, Acute Kidney Injury; BMI, Body Mass Index; CPB: cardiopulmonary by-pass; SOFA, Sepsis-related Organ Failure Assessment. Non-parametric comparison.

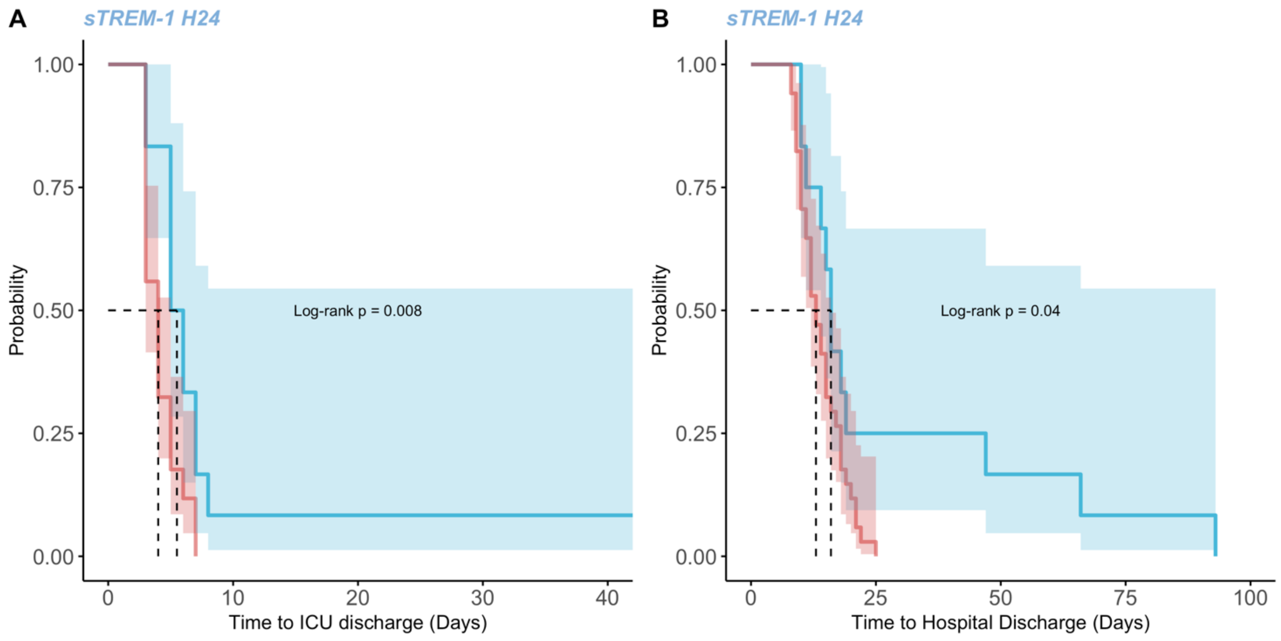

Supplementary Figure 5. Length of hospital and ICU stays between patients with low and high sTREM-1 levels. Estimation of Kaplan-Meir curves, representing the time to ICU and hospital discharge (days) between patients with low or high sTREM-1 levels (95% confidence interval). The curves are compared by a log-rank test. A high level of sTREM-1 is defined as a level higher than the third quantile in either cohort. **A**, time to ICU discharge between patients with high or low levels of sTREM-1 at H24. **B**: time to hospital discharge between patients with high or low levels of sTREM-1 at H24.

Supplementary Table 3

Parameters	AUC	CI	P.value	Threshold	Specificity	Sensitivity	Outcome
**TREM-1 H0**	0.73	0.57-0.89	0.009	191.0	0.56	0.83	Prolonged ICU stay
**TREM-1_H2**	0.72	0.56-0.89	0.011	243.5	0.50	0.83	
**TREM-1 H24**	0.73	0.56-0.9	0.009	304.5	0.68	0.83	
**TREM-1 variations H0_H2**	0.52	0.28-0.75	0.573	7.3	0.91	0.33	
**TREM-1 variations H0_H24**	0.57	0.33-0.81	0.240	95.5	1	0.33	
**TREM-1 variations H2_H24**	0.57	0.36-0.79	0.225	5.9	0.50	0.75	
**TREM-1_H0**	0.70	0.50-0.90	0.026	339.6	0.92	0.50	Prolonged hospital stay
**TREM-1_H2**	0.72	0.51-0.94	0.016	268.5	0.61	0.80	
**TREM-1 H24**	0.67	0.48-0.87	0.049	725	1	0.30	
**TREM-1 variations H0_H2**	0.56	0.35-0.78	0.286	65.3	0.89	0.30	
**TREM-1 variations H0_H24**	0.53	0.26-0.8	0.401	92.9	0.97	0.40	
**TREM-1 variations H2_H24**	0.48	0.23-0.73	0.567	21.8	0.69	0.50	
**TREM-1_H0**	0.71	0.53-0.89	0.010	325.8	0.90	0.53	Acute atrial fibrillation
**TREM-1_H2**	0.75	0.58-0.92	0.003	393.5	0.93	0.53	
**TREM-1 H24**	0.79	0.63-0.94	0.001	336.5	0.81	0.73	
**TREM-1 variations H0_H2**	0.53	0.31-0.75	0.381	47.5	0.81	0.47	
**TREM-1 variations H0_H24**	0.68	0.48-0.88	0.024	84.3	0.97	0.47	
**TREM-1 variations H2_H24**	0.69	0.5-0.87	0.021	29.1	0.84	0.53	
**TREM-1_H0**	0.83	0.66-1	0.003	325.8	0.87	0.86	Acute kidney injury at Day 2
**TREM-1 H2**	0.86	0.65-1	0.001	414.0	0.92	0.86	
**TREM-1_H24**	0.90	0.7-1	0.0004	541.0	1	0.86	
**TREM-1 variations H0_H2**	0.66	0.45-0.88	0.091	47.5	0.77	0.57	
**TREM-1 variations H0_H24.**	0.82	0.64-1	0.002	52.0	0.74	0.86	
**TREM-1 variations H2 H24.**	0.80	0.66-0.94	0.005	9.6	0.56	1	

Supplementary Table 3 : **Predictive value of TREM-1 levels** at H0, H2, H24 or TREM-1 variations for prolonged ICU stay, prolonged hospital stay, post-operative acute atrial fibrillation occurrence and post-operative acute Kidney injury occurrence. Best threshold for sensitivity and specificity was calculated by Youden index.
